# Challenges of diagnosing severe malaria with complications in adult patients: a case report

**DOI:** 10.1186/s40794-023-00216-7

**Published:** 2024-04-01

**Authors:** Rika Bur, Erni Juwita Nelwan, Ira Danasasmita, Gardian Lukman Hakim, Syukrini Bahri, Febby Elvanesa Sandra Dewi, Rana Zara Athaya, Leonard Nainggolan

**Affiliations:** 1 Division of Tropical and Infectious Diseases, Department of Internal Medicine, Faculty of Medicine, Universitas YARSI/YARSI Hospital, Jalan Letjen Suprapto No. Kav 13, Cempaka Putih Timur, Central Jakarta, Jakarta, 10510 Indonesia; 2 Division of Tropical and Infectious Diseases, Department of Internal Medicine, Faculty of Medicine, Universitas Indonesia/Dr Cipto Mangunkusumo Hospital, Jakarta, 10430 Indonesia; 3 YARSI Hospital, Jakarta, 10510 Indonesia; 4 Harapan Kita Women and Children Hospital, Jakarta, 11420 Indonesia

**Keywords:** Diagnostic challenge, Indonesia, Malaria, Plasmodium, Urban population

## Abstract

**Background:**

Malaria is known to be the main cause of death in malaria-endemic areas. The authors report a case of severe malaria in an adult with no history of travel from an endemic area with good outcomes after hospitalization.

**Case presentation:**

A 46-year-old man was brought to the Emergency Room (ER) because of fever and chills for 6 days. Complaints were accompanied by nausea and vomiting three times a day. The patient also experienced headaches, weakness, coughing, and a runny nose after two days of admission. The patient had no history of traveling from a malaria-endemic area. The patient was transferred from the Emergency Department (ED) to the High Care Unit (HCU), and during 1 day of intensive care at the HCU, there was a clinical deterioration characterized by dyspnea, icteric sclerae, acral edema, tenderness in both calves, and rash in the abdominal area. Due to worsening respiratory function, the patient was placed on a ventilator. During intensive treatment, the patient continued to show deterioration. The clinical findings suggested a possible feature of Weil’s disease or fulminant hepatitis, and although the patient was in intensive care, there was no clinically significant improvement. Furthermore, microscopic blood smear examination and rapid diagnostic tests (RDTs) for malaria were carried out on the 4th day of treatment with negative results. As there was no clinically significant improvement, it was decided to take a blood smear and repeat RDT on the twelfth day, which showed a positive result for falciparum malaria. Subsequently, artesunate was administered intravenously, and the patient’s condition began to improve with a negative parasite count the following day. The patient was discharged in good clinical condition on day 25 of treatment.

**Conclusion:**

Good quality malaria diagnostic techniques are essential to diagnose malaria. A timely diagnosis of malaria has the potential to save the patient. Because Jakarta is not a malaria endemic area, it was concluded that this case was an introduced malaria case.

## Introduction

According to the latest report from the WHO, it was estimated that there were 241 million cases of malaria and 627,000 deaths from malaria worldwide in 2020. This shows that the number of malaria cases in 2020 increased by as much as 14 million compared to 2019, and the number of deaths increased by 69,000. Two-thirds of these deaths were associated with barriers to the prevention, diagnosis, and treatment of malaria [1].

Severe, fatal malaria is caused primarily by *Plasmodium falciparum*. Management and prognosis depend on establishing the diagnosis in patients who have a history of travel from endemic areas, the ability of early detection, and effective and timely treatment. Severe malaria is defined as acute malaria with evidence of organ failure and/or a high degree of parasitemia, and this condition is associated with a high mortality rate [2]. Severe malaria is a medical emergency that requires immediate effective treatment to reduce the risk of death [3]. Microscopic examination of blood smears is still the gold standard for diagnosing malaria [4]; however, in certain conditions, such as patients with no history of travel to endemic areas and malaria screening procedures, the diagnosis of malaria with RDTs can become more difficult to confirmed. In this case report, severe malaria was diagnosed in an adult without a history of traveling from an endemic area with a good outcome after the patient received adequate malaria treatment.

According to the World Health Organization (WHO) imported malaria can be defined as a “malaria case or infection in which the infection was acquired outside the area in which it is diagnosed” meaning the malaria case diagnosis was confirmed within 3 months period of returning from an endemic area. Meanwhile, introduced malaria case refer to “malaria acquired by mosquito transmission from an imported case in an area where malaria is not a regular occurrence” [5]. Although it can occur in certain circumstances, transmission of malaria in nonendemic locations is extremely rare. Adverse effects such as an increased risk of death can occur in individuals with a delayed malaria diagnosis [6]. Thus, this patient more fit into the definition of introduced malaria cases.

### Case

**Fig. 1:**
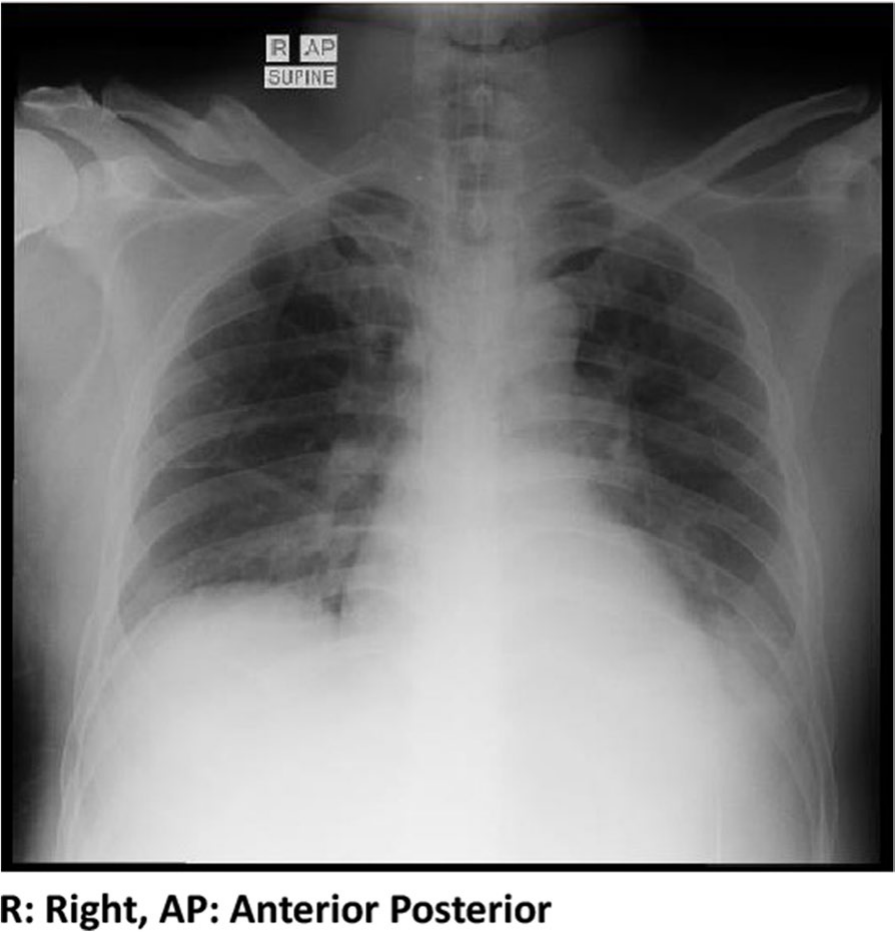
Chest X-ray showing bilateral pulmonary infiltrate with left pleural effusion

A 46-year-old man was brought to the Emergency Room at Yarsi Hospital, Jakarta, due to fever and chills starting 6 days prior. Complaints were accompanied by nausea and vomiting three times a day. The patient also experienced headaches, weakness, coughing, and a runny nose. The patient did not have a history of any diseases and had no history of travel to a malaria-endemic area before. The patient works as a motorcycle taxi driver. On presentation, vital signs were as follows: blood pressure of 100/65 mmHg, pulse of 126 beats per minute, respiratory rate of 36 times per minute, temperature of 37.6 °C, and 98% oxygen saturation with oxygenation of 5 l per minute via nasal cannula. Initial laboratory test results were as follows: hemoglobin 15.1 g/dL, leukocytes 7800/μL, platelets 51,000/μL, aspartate aminotransferase (AST) 172 U/L, alanine aminotransferase (ALT) 91.1 U/L, sodium 120 mmol/L, potassium 2.8 mmol/L, and chloride 87 mmol/L. Total bilirubin was 6.26 mg/dL, with direct bilirubin 4.90 mg/dL. The polymerase chain reaction (PCR) test for COVID-19 and the leptospirosis serological test were both negative. Chest radiograph showed bilateral opacity in the lung bases and a left pleural effusion (Fig. 1). The patient working as a motorcycle taxi driver in Jakarta which drives people from and to the airport.

Initially, the patient was admitted to the emergency room and then transferred to the HCU. During intensive medical treatment at the HCU, which lasted 1 day, there was clinical deterioration characterized by dyspnea, scleral icterus, acral edema, tenderness over both calves and an abdominal rash. Due to worsening respiratory condition, a ventilator was then installed. The patient also received penicillin G therapy of 1.5 million units four times per day, and electrolyte disturbances were corrected during the first day of treatment at the HCU. Further laboratory examination results obtained the following values: hemoglobin 14.4 g/dL, leukocytes 6000/μL, platelets 52,000/μL, urea 119 mg/dL, creatinine 1.34 mg/dL, albumin 2.7 g/dL, sodium 128 mmol/L, potassium 3.1 mmol/L, chloride 97 mmol/L and magnesium 3.3 mg/dL. Blood gas analysis showed pH 7.44, pO2 90 mmHg, pCO2 36 mmHg, HCO3 25 mmol/L, and base excess 1.8 mmol/L. Laboratory check for hepatitis such as detecting Hepatitis B surface antigen (HbsAg) and antibody for hepatitis C (anti-HCV), the results were negative.

On the fourth day, a malaria rapid test and peripheral blood smear were carried out, but the results were negative. On the seventh day of being admitted to the HCU, hypotension occurred, and it was decided to give adrenaline 0.12 mcg/kg/minute and vasopressin 0.04 IU/minute. On investigation of his hypotension, the laboratory results were as follows: AST 50.8 u/l, ALT 37.1 U/L, sodium 135 mmol/L, potassium 4.9 mmol/L, chloride 102 mmol/L, and albumin 2.4 g/dL, and leptospira serological test showed negative results.

**Fig. 2:**
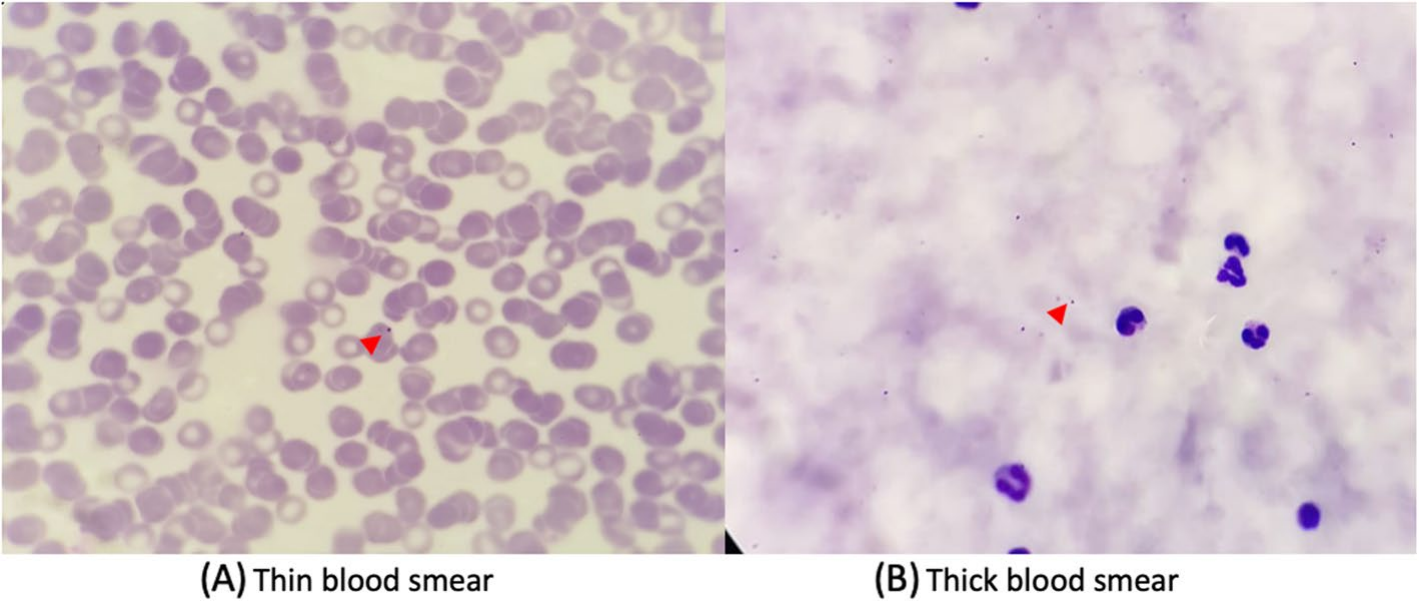
**a** Thin blood smear showed positive results for falciparum malaria trophozoite stage (**b**) Thick blood smear produced positive results for falciparum malaria trophozoite stage

**Fig. 3:**
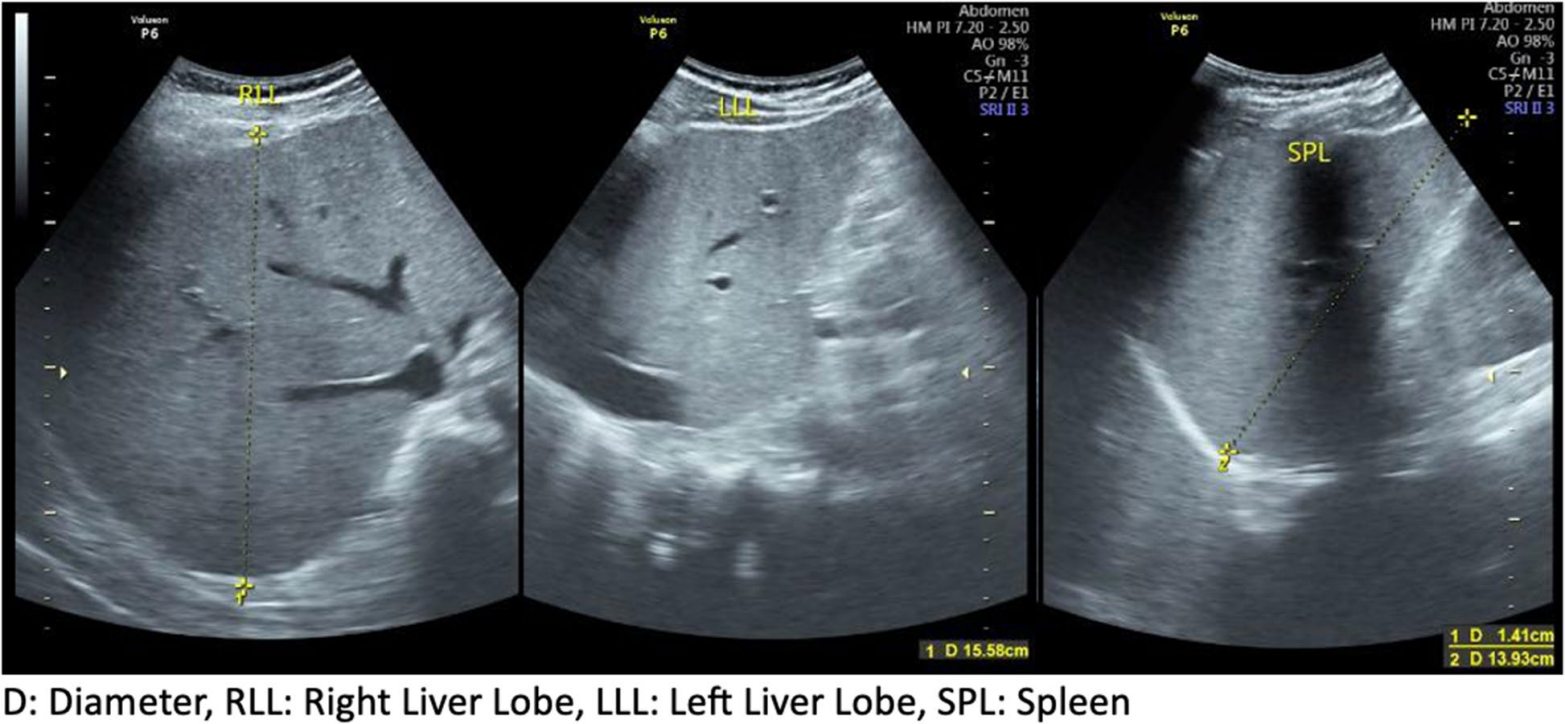
Abdominal sonography showing hepatosplenomegaly

On the twelfth day, repeat examination of peripheral blood and RDT for malaria was carried out, and the results were positive for both with a parasite count of 160 parasites/μL and a 5.3% level of parasitemia (Fig. 2). The Rapid Diagnostic Tools that being used was Right Sign Malaria Pf test (Biotest, Hangzhou Biotest Biotech Co, China) with both sensitivity and specificity was > 99.9%. Further, an ultrasound examination of the abdomen was performed and found hepatosplenomegaly (Fig. 3). Hemodialysis was also performed on day 12 in the HCU due to anuria and pulmonary edema. Chest X-ray evaluation showed increasing bilateral infiltrates. The broadening of therapy was determined based on the CXR result which leads to suspect of Pneumonia. The antibiotic choice to be added was ceftriaxone 1 × 2 gram. The patient also given artesunate intravenously on day 12 at a dose of 2.4 mg/kg body weight 3 times at 0, 12, and 24 hours on the first day and then 2.4 mg/kg body weight intravenously every 24 hours every day until the patient could tolerate oral medication. The number of parasites was counted after patient received artesunate therapy for two days, and both thick and thin blood smear shown no parasites can be identified from the patients sample.

After the patient was cooperative and able to take oral intake, he was given dihydroartemisinin-piperaquine (DHP) for 3 days and 1 tablet of primaquine for 1 day. The rationale for using primaquine is that is an 8-aminoquinoline drug that kills and sterilizes the mature *P. falciparum* gametocytes, and also it became a common standard and widely available regimen to combine this important gametocytocidal drug with conventional malaria treatment to prevention of *Plasmodium falciparum* malaria transmission especially in endemic region. Before DHP was administered, glucose-6-phosphate dehydrogenase (G6PD) screening was carried out and he was identified as not being G6PD deficient.

After malaria treatment, the patient’s clinical condition continued to improve, and laboratory evaluation showed a hemoglobin value of 10.9 g/dL, a platelet count of 431/μL, a leukocyte count of 11,300/μL (differential counts were basophils 0.47%, eosinophils 4.3%, neutrophils 82.0%, lymphocytes 8.3%, monocytes 5.0%), an AST of 55 U/L, and an ALT of 109.7 U/L. Repeat X-ray examination showed a decrease in the number of pulmonary infiltrates. The patient was then discharged after 25 days of treatment at the HCU.

## Discussion

In this case report, the authors report a case of severe malaria in an adult with no history of travel to an endemic area and was RDT/smear negative during acute presentation which found later to be positive on the twelfth day of treatment. More than 10,000 cases of entry of malaria from endemic to nonendemic countries are reported annually; however, cases of introduced malaria in malaria-free urban areas in malaria-endemic countries are less well known [7]. Local transmission causes malaria cases in nonendemic areas to occur in several conditions, including through 1) “airports”, 2) “harbors”, 3) “baggage” malaria, 4) nosocomial transmission, and 5) local transmission by malaria vectors. In addition, cases of mild malaria outbreaks due to local transmission were reported in northern Germany and Berlin until 1947 [8] and in the United States [9].

Transmission of the malaria parasite near airports can occur through infected Anopheles mosquitoes carried by aircraft, which can survive long-distance flights and adapt long enough to new environments [10]. Locally acquired malaria, where cases are mainly concentrated around international airports, is known as airport malaria [11]. The risk factor for this patient may be related to his work as a motorcycle taxi driver. Driving a vehicle in the area around the airport to pick up passengers can increase the patient’s risk of being bitten by Anopheles mosquitoes around the airport. There is ample evidence that airport malaria is on the rise in malaria-free countries. Between 2010 and 2020, the number of infected people in Europe increased 7.4 times compared to the decade of 2000-2009 [11]. This increase may be related to climate change, increased international trade, decreased aircraft disinfection, and delays in diagnosing and treating cases. More importantly, current interventions at preventing this spread are undermined by biological and operational challenges such as malaria parasite drug resistance and vector resistance to insecticides, as well as logistical limitations for instance due to the pandemic the delivery of antimalaria and RDT was hindered between and within countries further causing a lack of local stocks [11]. Therefore, there is a need to strengthen malaria prevention and treatment of people at risk of malaria at airports and to implement strict and regular entomological and epidemiological surveillance in and around airports.

Global warming has become undeniable, and its magnitude and impact on malaria transmission are increasing. For instance, the number of days with average temperatures above 25 °C in summer is increasing. On the other hand, airport malaria cases observed in the last decade were mainly associated with favorable climatic conditions for mosquito survival (mean temperature 23 °C, range: 17–31 °C) [12]. One of the key factors in mosquito development, both in water and on land, and in mosquito parasite development is temperature. Indeed, the duration of sporulation, life expectancy of mosquitoes, and duration of larval development are strongly influenced by temperature [13].

The decision to repeat laboratory testing for malaria was based on the following findings: 1) clinical deterioration continued even with intensive medical treatment, 2) indications of respiratory failure, 3) persistently elevated liver enzymes, 4) elevated levels of urea and creatinine, and 5) thrombocytopenia. Previous studies reported that respiratory failure occurs in 10-25% of cases of severe malaria caused by *P. falciparum* [14, 15]. According to CDC, patient can be diagnosed with severe malaria if fulfilled at least one of criteria including high percent parasitemia (> 5%), severe anemia (Hb < 7 g/dL), or metabolic abnormalities such as impaired consciousness, seizures, circulatory collapse/shock, acute respiratory distress syndrome (ARDS) or pulmonary edema, acidosis, acute kidney injury, disseminated intravascular coagulation (DIC) or abnormal bleeding, and jaundice [16]. Thus, despite the initial negative RDT and smear, the severe malaria diagnosis are still suspected with elevated serum aspartate and alanine aminotransferase levels, as the infection can cause liver dysfunction. The patient also showed a decrease in the number of platelets as well as an increase in the level of bilirubin which implicates liver dysfunction found in malaria cases [16].

Our center follows standardized laboratory and protocols within WHO standards. Malaria smear examination is being employed to identify the presence of malaria parasites in a patient’s blood. The process involves obtaining a small blood sample from the patient, usually from a fingertip, and creating two types of blood smears on microscope slides which are a thin smear and a thick smear. The thin smear is generated by spreading a small amount of blood across one slide and air-drying it, while the thick smear is produced on a second slide with a larger, thicker distribution of blood. The thin smear is often fixed using heat or methanol. Both smears are then stained, typically with Giemsa stain, following specific staining protocols. Subsequently, the stained thin smear is examined under oil immersion on a microscope, allowing for the identification of malaria parasites and determination of the species present. The results, detailing the type of malaria parasite and the level of infection, are recorded, and reported [17].

The Rapid Diagnostic Tool that was being used was the Right Sign Malaria Pf test (Biotest, Hangzhou Biotest Biotech Co, China). For the RDT that we used the sensitivity and specificity reached> 99.9% which shows the most likely cause of false negative results was due to sample error including how the sample was stored and delivered. As with the limitation of diagnostic tests in general, the result should be interpreted together with other clinical information that has been required by the physician. Further, if negative results are accompanied by clinical symptoms, additional testing with alternative clinical methods is recommended. This RDT has several limitations in which it can only be used for in vitro diagnostic use only, is used for the detection of Plasmodium falciparum (P.f), Plasmodium vivax (P.v), Plasmodium ovale (P.o), and Plasmodium malariae (P.m.) specimens only which can be used for qualitative not quantitative purpose. Also, the result of RDT cannot be used as the sole criterion in diagnosing of malaria [18].

Regarding how the RDT works, firstly whole blood (10 μL) sample from the patient was added into a specific tube and then mixed with three drops of buffer. After that, the test strip was vertically inserted into the previous tube and then we were able to see results within 10 min. The interpretation of the result depended on the band’s appearance. The appearance of two-colored bands which refer to control line ‘C’ along with test line ‘T’) can be inferred as a P. falciparum-positive result. On the other hand, if only a single-colored line on the control line ‘C’ is interpreted as negative results. Results were deemed invalid if any color could not be found on the control line [19].

False-negative (FN) examination findings can result from the procurement and use of poor-quality RDTs, issue with the microscope’s quality, or varied capability between lab worker in reading and interpret smears [20]. Another possibility is that poor storage procedures for RDTs, as well as prolonged exposure to high temperatures, could affect their diagnostic efficacy. Although rare, operator error in checking and/or interpreting the RDT can lead to false negative results [21]. One of the causes of FN-RDT results that has received recent attention is deletion of the pfhrp2/3 genes [22]. These deletions have been reported initially in South America and now in several locations in Asia and Africa [23]. Parasites with these gene deletions are not recognized by PfHRP2-based RDTs, leading to the prediction that RDTs expressing only PfHRP2 recognize select parasites [24].

Based on the study conducted by Ditombi et al. which compare four malaria RDT, more than 95% of positive blood smear were confirmed positive based on the RDT test. In addition, the number of false-positive results were ranging between 14.1 -17.5% depending on the specific RDTs. Further the number of false-negative results were less than 5% of febrile cases of total population [19]. For the RDT that we used the sensitivity and specificity was reaching > 99.9% which shows the most likely cause of false negative results was due to sample error including how the sample was stored and delivered.

Chest X-ray examination showed bilateral pleural effusion and pulmonary infiltrates. Therefore, early ventilator intervention was carried out in these patients. Adults with severe *P. falciparum* malaria are at risk of developing noncardiogenic pulmonary edema and acute respiratory distress syndrome (ARDS), which have a high mortality rate. This is very common in patients infected with *P. falciparum*. ARDS can occur in 5-25% of people with severe falciparum malaria [25]. Although rare, severe malaria must be differentiated from fulminant liver failure due to viral infection. This can have implications in determining treatment and patient outcomes. In patients with severe malaria, the liver can be affected to varying degrees from liver dysfunction to heme abnormalities such as anemia. Also, in patients with severe malaria infection, the incidence of jaundice was 2.58%. The presence of jaundice in falciparum malaria indicates a severe condition of the disease with a higher incidence of complications [26]. Another differential diagnosis in this case was Weil’s disease due to pathognomonic signs of gastrocnemius muscle pain. In addition, pulmonary involvement can occur (20-70%). However, the symptoms are often mild without complications. The incidence of ARDS in malaria requiring mechanical ventilation is rare, but when it occurs, the mortality rate from ARDS can reach 50%. Such conditions are often associated with pulmonary hemorrhage due to disruption of the vascular endothelium and impaired coagulation [27].

## Conclusion

The route of transmission of severe *P. falciparum* malaria in Jakarta, which is rare, is not known. One of the possible explanations was that climate change increases Anopheles vectors, and transportation systems between regions or countries greatly affect the incidence of malaria in nonendemic areas, which further increases the chances of introduced case of malaria. Jakarta is a malaria-free area, and the patient has never been to a malaria-endemic area, and the patient’s work as a motorbike taxi driver at the international airport area allows him to come into contact with Anopheles mosquitoes brought by passengers from malaria endemic areas. Overall, better diagnostic techniques that avoid false negative findings and adequate training of laboratory personnel are essential to create timely diagnoses and further enable sufficient management of malaria cases.

## Data Availability

Data sharing is not applicable to this article as no datasets were generated or analysed during the current study.

## Abbreviations

HCU: High care unit
AST: Aspartate aminotransferase
ALT: Alanine aminotransferase
G6PD: Glucose-6-Phosphate Dehydrogenase
